# Targeting the TBK1-p62 condensate axis restores sensitivity to EGFR-TKIs in resistant lung cancer

**DOI:** 10.1093/procel/pwag043

**Published:** 2026-06-26

**Authors:** Yuexiao Song, Changchun Shao, Yiruo Zhang, Min Yang, Yaqian Liu, Sheng Chen, Jia Gao, Zihuan Li, Bin Li, Xuebiao Yao, Xing Liu, Ke Ruan, Yingying Du

**Affiliations:** Department of Oncology, The First Affiliated Hospital of Anhui Medical University, Hefei 230022, China; Department of Oncology, The First Affiliated Hospital of Anhui Medical University, Hefei 230022, China; Department of Oncology, The First Affiliated Hospital of Anhui Medical University, Hefei 230022, China; The First Clinical Medical College, Anhui University of Chinese Medicine, Hefei 230022, China; Foresight Therapeutics (Hefei), Co., Ltd, Hefei 230022, China; Department of Oncology, The First Affiliated Hospital of Anhui Medical University, Hefei 230022, China; Department of Oncology, The First Affiliated Hospital & School of Life Sciences, Ministry of Education Key Laboratory for Membrane-less Organelles & Cellular Dynamics, Hefei National Research Center for Interdisciplinary Sciences at the Microscale, Biomedical Sciences and Health Laboratory of Anhui Province, Center for Advanced Interdisciplinary Sciences and Biomedicine of IHM, Division of Life Sciences and Medicine, University of Science and Technology of China, Hefei 230022, China; Department of Oncology, The First Affiliated Hospital & School of Life Sciences, Ministry of Education Key Laboratory for Membrane-less Organelles & Cellular Dynamics, Hefei National Research Center for Interdisciplinary Sciences at the Microscale, Biomedical Sciences and Health Laboratory of Anhui Province, Center for Advanced Interdisciplinary Sciences and Biomedicine of IHM, Division of Life Sciences and Medicine, University of Science and Technology of China, Hefei 230022, China; Foresight Therapeutics (Hefei), Co., Ltd, Hefei 230022, China; Department of Oncology, The First Affiliated Hospital & School of Life Sciences, Ministry of Education Key Laboratory for Membrane-less Organelles & Cellular Dynamics, Hefei National Research Center for Interdisciplinary Sciences at the Microscale, Biomedical Sciences and Health Laboratory of Anhui Province, Center for Advanced Interdisciplinary Sciences and Biomedicine of IHM, Division of Life Sciences and Medicine, University of Science and Technology of China, Hefei 230022, China; Department of Oncology, The First Affiliated Hospital & School of Life Sciences, Ministry of Education Key Laboratory for Membrane-less Organelles & Cellular Dynamics, Hefei National Research Center for Interdisciplinary Sciences at the Microscale, Biomedical Sciences and Health Laboratory of Anhui Province, Center for Advanced Interdisciplinary Sciences and Biomedicine of IHM, Division of Life Sciences and Medicine, University of Science and Technology of China, Hefei 230022, China; Department of Oncology, The First Affiliated Hospital & School of Life Sciences, Ministry of Education Key Laboratory for Membrane-less Organelles & Cellular Dynamics, Hefei National Research Center for Interdisciplinary Sciences at the Microscale, Biomedical Sciences and Health Laboratory of Anhui Province, Center for Advanced Interdisciplinary Sciences and Biomedicine of IHM, Division of Life Sciences and Medicine, University of Science and Technology of China, Hefei 230022, China; Department of Oncology, The First Affiliated Hospital of Anhui Medical University, Hefei 230022, China; Department of Oncology, The Fifth Affiliated Hospital of Anhui Medical University, Fuyang 236000, China

**Keywords:** EGFR-TKI resistance, lung cancer, liquid-liquid phase separation (LLPS), biomolecular condensates, SQSTM1/p62, TBK1

## Abstract

Acquired resistance to epidermal growth factor receptor tyrosine kinase inhibitor (EGFR-TKI) in non-small cell lung cancer remains a pressing clinical challenge. Liquid-liquid phase separation has emerged as a new mechanism of drug resistance, yet its role in EGFR-TKI resistance in lung cancer is largely unexplored. Herein, we prioritized Sequestosome 1 (SQSTM1/p62) as a key condensate by integrating proteomics data from EGFR-TKI resistance cell lines and clinical biopsy specimens, in which the cytoplasmic p62 condensate formation positively correlated with EGFR-TKI resistance. Domain mapping demonstrated that the PB1 and UBA domains of p62 were critical for promoting phase separation and reducing sensitivity to EGFR-TKI treatment, whereas S403 phosphorylation promoted p62 condensation and EGFR-TKI resistance. Further xenograft studies validated that reduction of p62 condensation restores EGFR-TKI sensitivity. Kinase enrichment and interaction assays identified TBK1 as an upstream regulator of p62 S403 phosphorylation to promote p62 condensation after pharmacological inhibition of EGFR. Notably, among the five compounds identified from a drug library screen that both disrupted p62 condensate formation and inhibited the viability of resistant cells, the highly selective TBK1 inhibitor GSK8612 stood out. GSK8612 inhibited p62 S403 phosphorylation and consequently, at subcytotoxic doses, synergized with EGFR-TKIs to suppress the viability of resistant cells and tumor growth in the xenograft mouse model. These findings propose TBK1-p62 axis links p62 condensate homeostasis to EGFR-TKI resistance as an underlying mechanism of action and an emerging strategy to resensitize EGFR-TKI treatment in resistant lung cancer.

## Introduction

Lung cancer ranks first in incidence and mortality among all malignancies (Bray et al., 2024). A substantial subset of patients with non-small cell lung cancer (NSCLC), particularly those with lung adenocarcinoma and patients from Asian populations, harbor epidermal growth factor receptor (EGFR) driver mutations (Soo et al., 2024). As the standard treatment for these patients, the EGFR tyrosine kinase inhibitors (EGFR-TKIs) have evolved to third ­generation, e.g., Osimertinib, which extends median progression-free survival to 18–20 months (Soria et al., 2018). However, almost all patients ultimately acquire resistance to EGFR-TKIs, and the limited therapeutic options after resistance develops lead to substantially shorter overall survival (Leonetti et al., 2019). Accordingly, clarifying the molecular basis of EGFR-TKI resistance and developing intervention strategies for lung cancer is of critical significance.

Emerging evidence has implicated biomolecular condensates formed via liquid-liquid phase separation (LLPS) as regulators of resistance to chemotherapy, immunotherapy, and endocrine therapy (Wei et al., 2023; Xie et al., 2022; Yu et al., 2021). Biomolecular condensates driven by multivalent interactions serve as novel molecular hubs for key cancer-related cellular processes (Ruan et al., 2024; Shin and Brangwynne, 2017; Song et al., 2023), e.g., super-enhancer-driven transcription, DNA damage responses, signal transduction, and RNA metabolism and splicing (Esposito et al., 2021; Kilic et al., 2019; Li et al., 2020; Sabari et al., 2018). Dysregulation of LLPS homeostasis is hence closely associated with tumorigenesis, progression, metastatic dissemination, and resistance to therapy (Deng et al., 2026; Liu et al., 2023; Lu et al., 2021). Pharmacological intervention targeting biomolecular condensates enables simultaneous perturbation of multiple components through cascade effects (Fan et al., 2024; Yu et al., 2021), thus paving a new path to targeting those conventionally deemed undruggable. A few successful cases have demonstrated modulation of phase separation as an effective approach against chemotherapy resistance (Tang et al., 2025; Zhan et al., 2024). Therefore, LLPS holds promise for providing novel insights into the mechanisms and interventions of drug resistance in EGFR-TKI-resistant lung cancer.

In this study, we integrated clinical lung cancer biopsies and EGFR-TKI-resistant cells to interrogate condensate formation and EGFR-TKI resistance. Proteomics and LLPS scoring prioritized Sequestosome 1 (SQSTM1/p62) as a key promoter of p62 droplet formation in resistant tissues and cells. The Phox and Bem1p domain (PB1) and ubiquitin-associated domain (UBA) domains of p62 govern condensate formation and modulate sensitivity to EGFR-TKI treatment. Functionally, p62 knockout (p62-KO)/overexpression in mice xenografts collectively supported a positive association between p62 and EGFR-TKI resistance. Several drugs were repurposed to modulate p62 droplet formation, including a selective TANK-binding kinase 1 (TBK1) inhibitor GSK8612. Coincidentally, TBK1 was identified as an upstream regulator of p62 condensates through phosphorylation of p62 at the S403 residue. Pharmacological inhibition using GSK8612 (Thomson et al., 2019) reduced p62 S403 phosphorylation, suppressed p62 condensation, and restored sensitivity to EGFR-TKIs *in vitro* and *in vivo*. Collectively, our work identifies the TBK1-p62 axis as a therapeutically tractable condensate-centric vulnerability, i.e., EGFR-TKI-resistant cells rely on TBK1-driven p62 condensate formation, thus providing a potential strategy to pharmacologically modulating LLPS to overcome EGFR-TKI resistance in lung cancer.

## Results

### Enhancement of p62 condensation in the EGFR-TKI-resistant lung cancer

We performed proteomic analysis on biopsy specimens from three treatment-naïve patients with EGFR driver mutations and three patients who developed resistance after EGFR-TKI therapy (Fig. 1A and Table S1). We reviewed the existing literature and found that multiple proteins are upregulated in EGFR-TKI-resistant cells (Qi et al., 2021). Notably, 16 proteins were consistently upregulated in both EGFR-TKI-resistant cells and clinical biopsy tissues (Fig. 1B). The droplet-promoting regions of these proteins were further scored using Predictor of Natural Disordered Regions (PONDR) and FuzDrop (Hatos et al., 2022; Xue et al., 2010). Notably, p62, sarcolemma-associated protein (SLMAP), and Ribosomal protein L7 (RPL7) harbor the highest LLPS capability (Fig. 1C), which have recently been confirmed experimentally (Tani et al., 2023; Turco et al., 2021; Wang et al., 2022). Immunofluorescence staining of p62 in biopsy samples (Fig. 1D) showed a large increase in p62 condensate formation following the development of EGFR-TKI resistance (Fig. 1E). These observations point to a potential role of p62 condensation in EGFR-TKI-resistant lung cancer.

**Figure 1.**
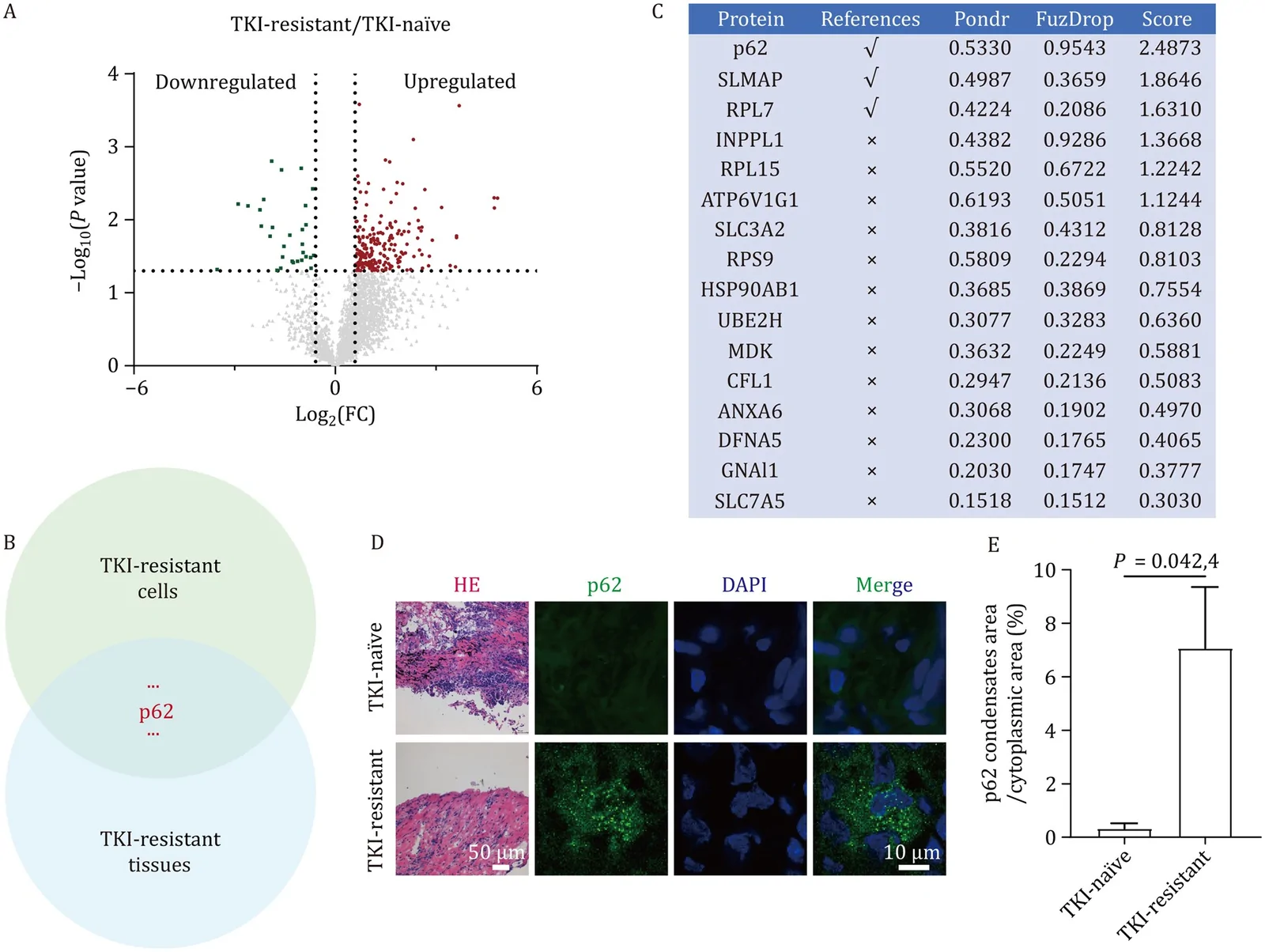
**Enhancement of p62 condensation in the EGFR-TKI-resistant lung cancer**. (A) Volcano plot shows differentially expressed proteins with (fold change, FC > 1.5) between tumor tissues before treatment (EGFR-TKI-naïve) and after acquisition of resistance (EGFR-TKI-resistant). (B) Venn diagram of proteins commonly increased in both cells and tissues after EGFR-TKI resistance. The cell-derived dataset was obtained from the publicly available Synapse database under accession ID syn25915977. (C) Phase separation capability of proteins upregulated in both cells and tissues after EGFR-TKI resistance. (D) Immunofluorescence of condensates stained by p62 antibody in biopsy samples before and after EGFR-TKI resistance. (E) Statistics of p62 condensate area across at least 9 cells in TKI-naïve and resistant biopsies. *n *= 3 biological replicates. Data are mean ± SEM; statistical analysis was performed using a two-tailed Student’s *t*-test (E).

### p62 condensates contribute to EGFR-TKI resistance in lung cancer

To elucidate the relationship between p62 condensates and EGFR-TKI resistance in lung cancer, we constructed Osimertinib-resistant PC9 (PC9 OsiR) and HCC827 (HCC827 OsiR) cells. PC9 OsiR and HCC827 OsiR (OsiR) (Fig. S1A and S1B), together with Gefitinib-resistant PC9 cells (PC9 GefR, GefR) (Fig. S1C), exhibited resistance indices (RI) >10, consistent with a highly drug-resistant phenotype. Gene sequencing suggested no additional EGFR mutations in the EGFR-TKI-resistant cells relative to the parental cells (Fig. S1D), indicating an alternative mechanism of drug resistance. Similar to biopsy samples obtained after EGFR-TKI resistance, the number and size of p62 condensates were significantly increased 3.7-, 6.6-, and 6.7-fold in PC9 OsiR, HCC827 OsiR and PC9 GefR cells (Figs. 2A, 2B, S2A and S2B). The enhanced green fluorescent protein (EGFP)-tagged p62 may fuse into larger droplets over time in PC9 and PC9 OsiR cells (Fig. 2C; Videos S1 and S2), while the diffusion speed of p62 condensates in resistant cells is much slower than in parental cells as revealed by the fluorescence recovery after photobleaching (FRAP) assay (Fig. 2D and 2E). These experiments validated that p62 forms condensates through LLPS in EGFR-TKI-resistant lung cancer.

**Figure 2.**
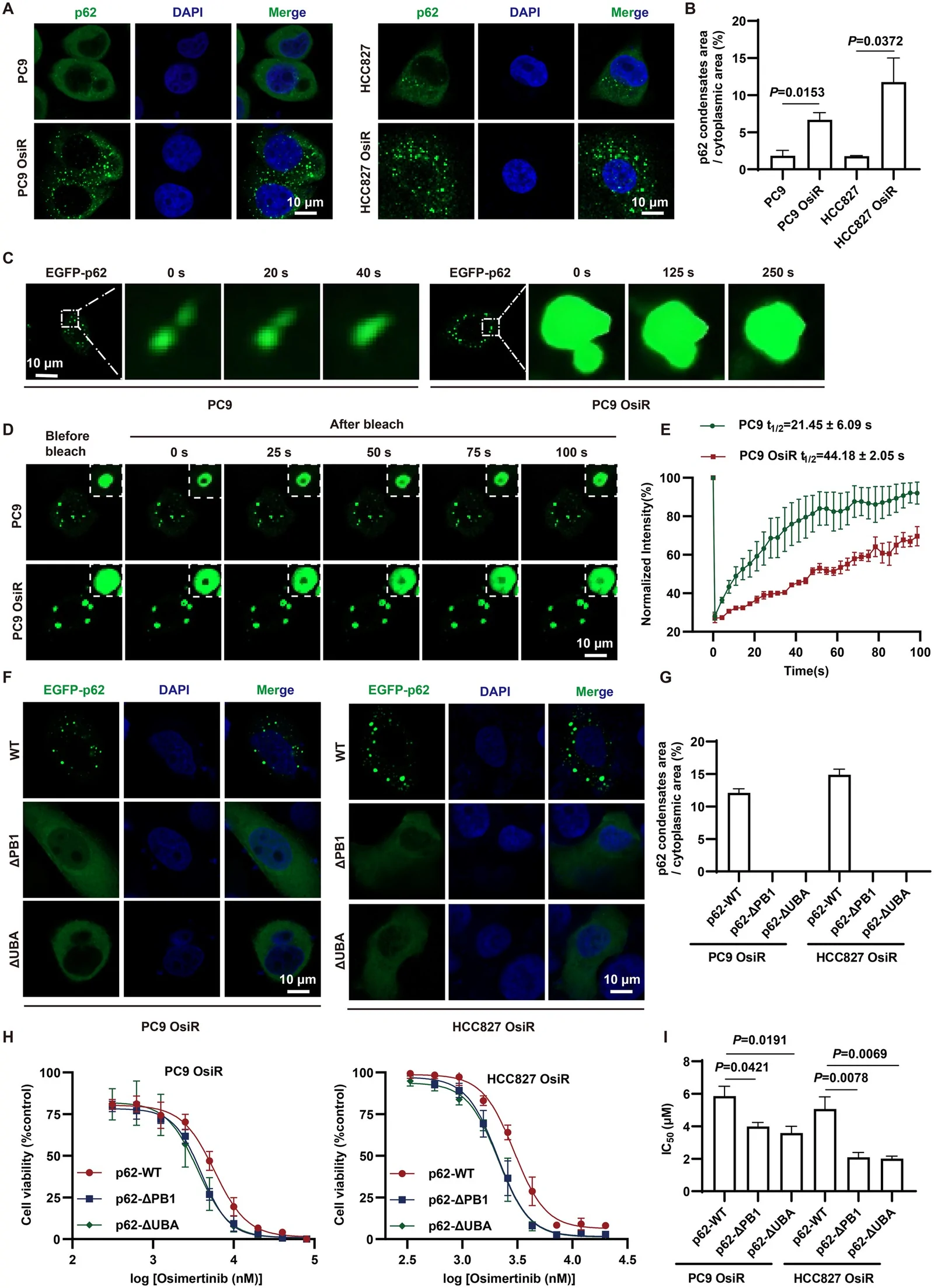
**p62 condensates contribute to EGFR-TKI resistance in lung cancer**. (A) Immunofluorescence of p62 condensates in PC9, HCC827, and OsiR cells. (B) The p62 condensate area as a percentage of the cytoplasmic area (quantified across > 9 cells). *n *= 3 biological replicates. (C) Time-lapse micrographs of EGFP-tagged p62 puncta fusion in PC9 and PC9 OsiR living cells. (D) Fluorescence recovery after photobleaching of p62-EGFP puncta in PC9 and PC9 OsiR cells. (E) The fluorescence intensities of 3 EGFP-p62 droplets after ­photobleaching. *n *= 3 biological replicates. (F) EGFP-p62 condensates in p62-KO OsiR cells rescued by wild-type or PB1/UBA domain-deleted p62. (G) The p62 condensate area as a percentage of cytoplasmic area (quantified across > 9 cells). *n *= 3 biological replicates. (H) CellTiter-Glo assay of cell viability in p62-KO OsiR cells rescued by either wild-type or domain-deleted p62, after 72 h treatment of Osimertinib. *n *= 3 biological replicates. (I) IC_50_ values of Osimertinib in OsiR cells. *n *= 3 biological replicates. Data are mean ± SEM; ­statistical analysis was performed using two-tailed Student’s *t*-test (B) or 1-way ANOVA (I).

To further dissect which domain of p62 was associated with droplet formation and EGFR-TKI resistance, we reconstituted p62 variants in p62-KO EGFR-TKI-resistant cells (Fig. S2C). Deletion of the PB1 (p62-ΔPB1) or UBA (p62-ΔUBA) domain suppressed the p62 condensation (Figs. 2F, 2G, S2D and S2E) relative to the wild-type p62 (p62-WT) in either OsiR or GefR cells. Previous studies at the level of *in vitro* purified proteins have also confirmed that the PB1 domain and UBA domain are essential for p62 phase separation (Sun et al., 2018). Notably, deletion of either the PB1 or the UBA domain rendered the resistant cells >1.5-fold more sensitive to EGFR-TKIs (Figs. 2H, 2I, S2F and S2G).

### The p62 protein is a key mediator of EGFR-TKI resistance in lung cancer

As phase separation is a concentration-dependent process (Hu et al., 2025), we evaluated the p62 expression levels before and after EGFR-TKI resistance. In the resistant cell lines PC9 OsiR, HCC827 OsiR and PC9 GefR, p62 protein expression was 1.5-, 1.3-, and 1.6-fold higher than in their parental counterparts, respectively (Figs. 3A, 3B, S3A and S3B). In lung cancer biopsy specimens obtained after the acquisition of EGFR-TKI resistance, p62 protein expression was increased by 14.5-fold relative to the pre-resistance specimens (Fig. 3C and 3D). To determine the functional contribution of p62 to the EGFR-TKI-resistant phenotype, we performed growth curve assays in three resistant cell models (Fig. S3C). Compared with control resistant cells, stable knockout of p62 markedly enhanced the sensitivity of resistant cells to EGFR-TKIs, reducing the half-maximal inhibitory concentration (IC_50_) values from 6.2 to 2.5 μmol/L in PC9 OsiR cells, from 2.8 to 1.1 μmol/L in HCC827 OsiR cells, and from 13.1 to 7.3 μmol/L in PC9 GefR cells (Figs. 3E, 3F, S3D and S3E). The plate colony formation assay similarly showed that p62-KO inhibits long-term colony formation of resistant cells and enhances the inhibitory effect of EGFR-TKIs (Fig. S3F and S3G). Conversely, overexpression of p62 in resistant cells decreased their sensitivity to EGFR-TKIs (Figs. 3G, 3H, and S3H–J), further supporting a functional role of p62 in maintaining the drug-resistant phenotype. To assess the role of p62 in mediating EGFR-TKI resistance *in vivo*, we injected PC9 OsiR cells or PC9 OsiR-p62-KO cells into nude mice, which then received vehicle or Osimertinib (2.5 mg/kg/day, oral gavage) (Fig. 3I). Clearly, the p62-KO promoted the antitumor effect of Osimertinib treatment, as evidenced by reduced tumor size in mice xenografted with p62-depleted PC9 OsiR cells (Fig. 3J). Regardless of treatment (vehicle or Osimertinib), tumors in p62-intact mice were more than 2-fold larger than those in p62-KO mice (Fig. 3K). These results underpin p62 as a key mediator of EGFR-TKI resistance in lung cancer.

**Figure 3.**
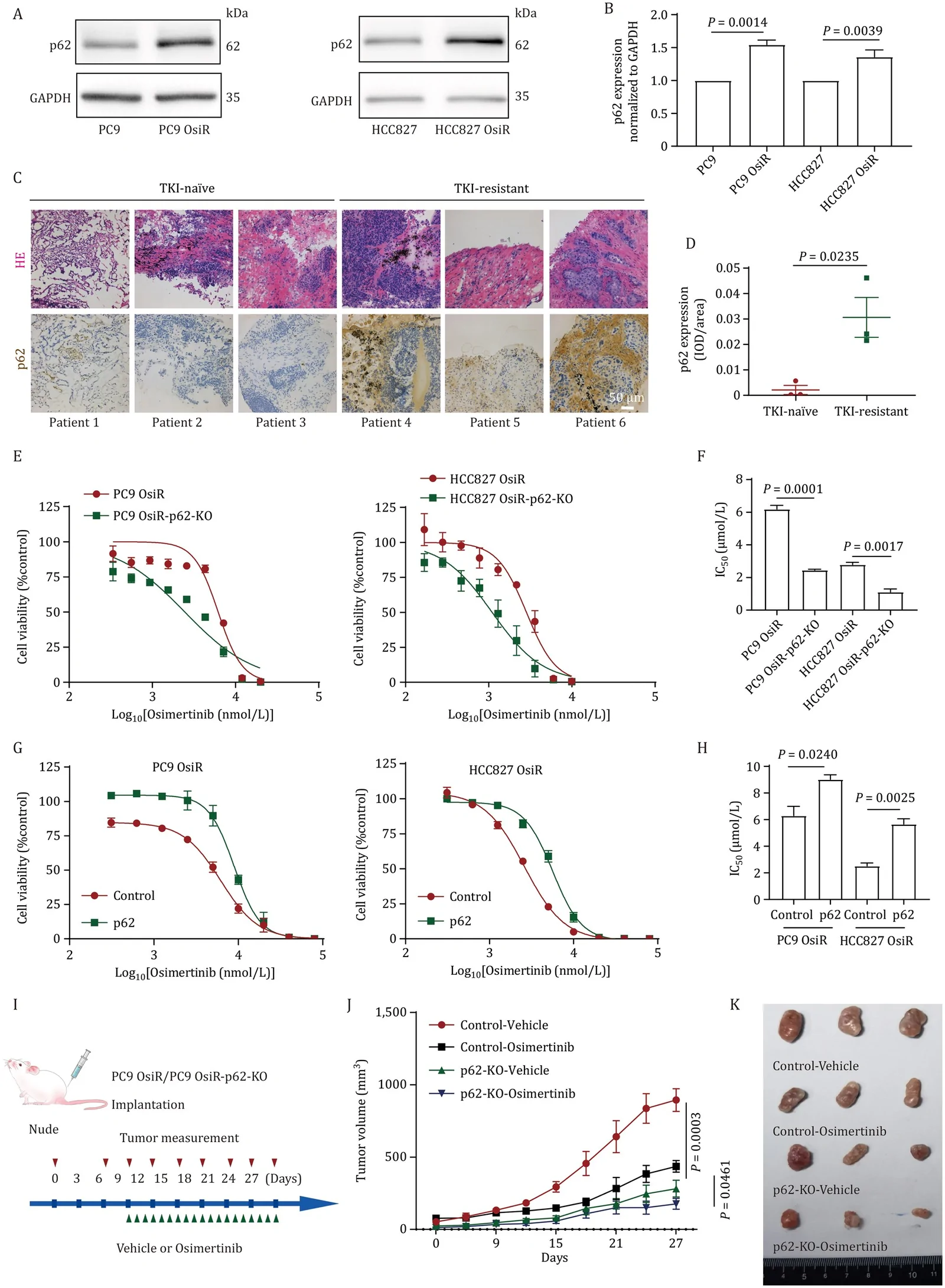
**The p62 protein is a key mediator of EGFR-TKI resistance in lung cancer**. (A) Western blot of p62 in parental or Osimertinib-resistant cells. (B) The statistics of the p62 protein expression level. *n *= 3 biological replicates. (C) The hematoxylin-eosin staining (HE) and p62 immunohistochemistry (IHC) in lung cancer biopsies obtained before treatment and after the development of EGFR-TKI resistance. (D) The statistics of the p62 expression level in TKI-naïve or resistant lung cancer biopsies. *n *= 3 biological replicates. (E) CellTiter-Glo assay of cell viability in p62 KO OsiR cells after 120 h treatment of Osimertinib. *n *= 3 biological replicates. (F) IC_50_ values of Osimertinib in OsiR cells. *n *= 3 biological replicates. (G) CellTiter-Glo assay of cell viability in p62 overexpression OsiR cells after 72 h treatment of Osimertinib. *n *= 3 biological replicates. (H) IC_50_ values of Osimertinib in OsiR cells. *n *= 3 biological replicates. (I) PC9 OsiR or PC9 OsiR-p62-KO cells (3.5 × 10^6^) were subcutaneously inoculated into nude mice for generating xenograft tumors. When tumors reached 100 mm³, mice were randomized into four groups treated for 19 days with: (i) vehicle control (2% DMSO + 40% PEG300 + 5% Tween80 + 53% saline), or (ii) Osimertinib (2.5 mg/kg/day, oral gavage). (J) The volume of the tumors was measured on the indicated days. *n *= 3 biological replicates. (K) At the end of the experiment, the tumors were dissected and imaged as indicated. Data are mean ± SEM; statistical analysis was performed using two-tailed Student’s *t*-test (B, D, F, H) or 2-way ANOVA (J).

To determine whether the autophagy-lysosome pathway is altered in Osimertinib-resistant cells, we examined autophagic flux, acidic lysosomal compartments, and Cathepsin D (CTSD) maturation in parental and EGFR-TKI-resistant cells. Compared with parental cells, both PC9 OsiR and HCC827 OsiR cells showed significantly increased bafilomycin A1 (BafA1)-induced p62 accumulation (Fig. S4A and S4B) and microtubule-associated protein 1 light chain 3 (LC3)-II turnover (Fig. S4C and S4D), indicating enhanced autophagic flux besides the autophagosome accumulation. Furthermore, EGFR-TKI-resistant cells exhibited markedly increased LysoSensor and LysoTracker signals, along with an enhanced punctate distribution in the perinuclear region (Fig. S4E and S4F). Compared with parental cells, resistant cells displayed decreased pro-CTSD and increased mature CTSD levels (Fig. S4G and S4H), resulting in a significantly elevated mature CTSD/pro-CTSD ratio (Fig. S4I). These findings indicate activation of the autophagy-lysosome pathway during acquired EGFR-TKI resistance, as evidenced by the expanded acidic lysosomal compartments, enhanced autophagic flux and CTSD maturation in EGFR-TKI-resistant cells.

### Screening of chemical modulators of p62 condensation in EGFR-TKI resistance lung cancer

To identify chemical modulators of p62 droplet formation, we treated PC9 OsiR cells with our in-house library of investigational or approved drugs (Fig. 4A). To improve throughput, cocktails of ten compounds each were added to PC9 OsiR cells to quantify the area of p62 condensates, using 1,6-hexanediol as the positive control for condensate disruption and dimethyl sulfoxide (DMSO) as a negative control. Notably, six cocktails inhibited p62 condensate formation, whereas three cocktails promoted it (Fig. S5A). Each component of these cocktails was then used individually to treat PC9 OsiR cells; 10 compounds were thus identified that suppressed p62 condensation, while two compounds robustly promoted p62 condensate formation (Figs. 4B, S5B and S5C). The cellular viability assay further identified tubercidin, chlorprothixene, menadione bisulfite sodium, vitamin K4, and GSK8612 as inhibitors of both PC9 OsiR cell growth and p62 condensate formation (Fig. 4C).

**Figure 4.**
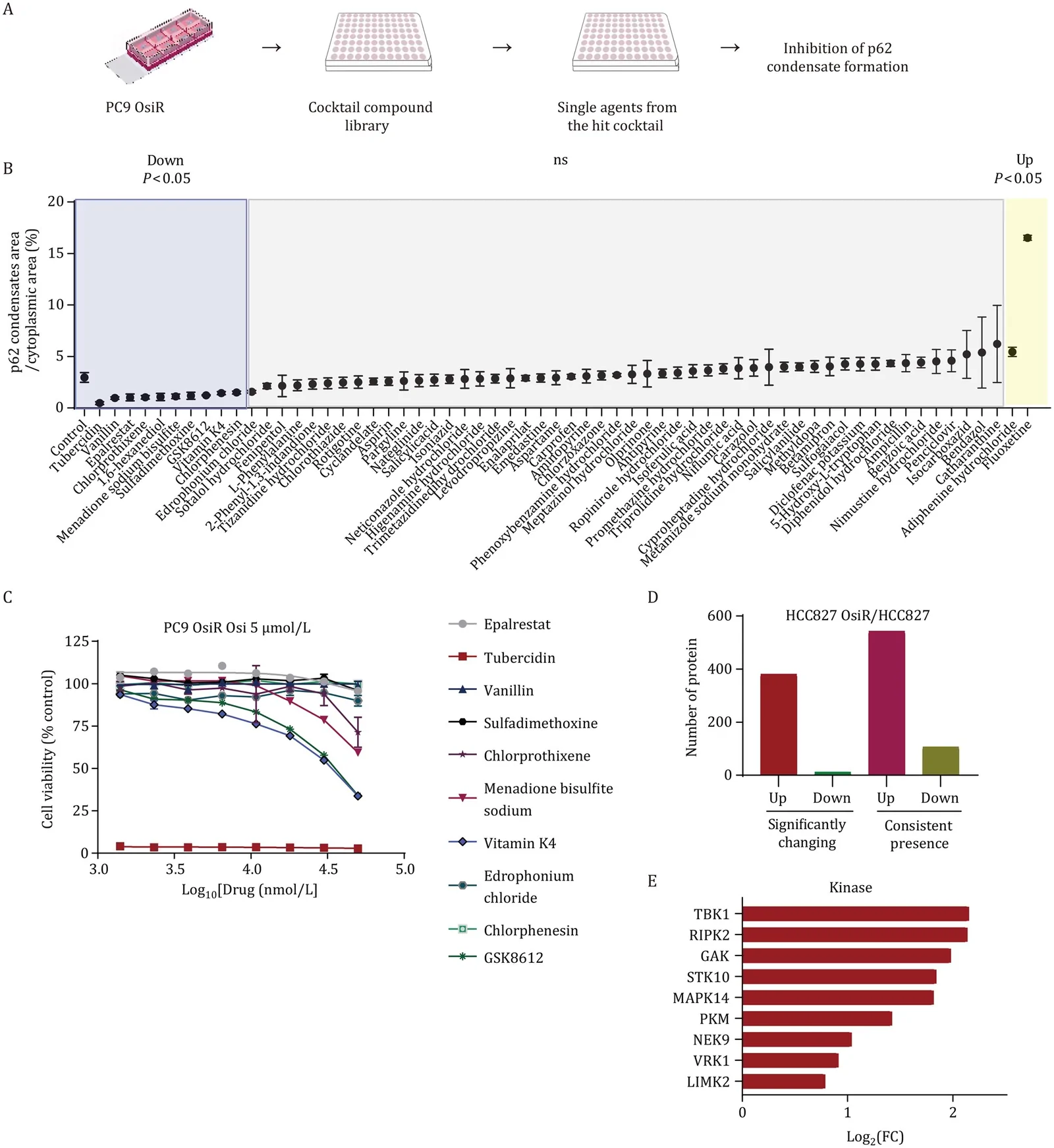
**Screening of chemical modulators of p62 condensation in EGFR-TKI-resistance lung cancer**. (A) Screening scheme of clinical compounds that modulate p62 LLPS in PC9 OsiR cells. (B) The effects of different compounds (50 μmol/L, 3 h) on the area of p62 condensates in PC9 OsiR cells. ns, not significant (quantified across > 3 cells). *n *= 3 technical replicates. (C) CellTiter-Glo assay of cell viability in PC9 OsiR cells after 72 h treatment of the identified p62 condensate inhibitors. *n *= 3 technical replicates. (D) The mass spectrometry analysis of proteins interacting with biotinylated p62 in HCC827 and HCC827 OsiR cells. (E) Kinase enrichment analysis of p62-interacting proteins. Data are mean ± SEM; statistical analysis was performed using two-tailed Student’s *t*-test (B).

To uncover the molecular targets of these five compounds in p62 condensates, the streptavidin magnetic beads were deployed to pull down proteins interacting with biotin-tagged p62 in HCC827 and HCC827 OsiR cells. Notably, 543 proteins were detected only in the resistant cells, whereas 107 proteins were present only in the parental cells (Fig. 4D). In comparison with the parental cells, 381 proteins were significantly upregulated, and 12 were downregulated in the HCC827 OsiR cells. The enrichment of more proteins into p62 condensates in Osimertinb resistant cells may enhance the multivalent interactions and reduce the intra-condensate diffusion rate of p62, as observed in the FRAP assays (Fig. 4D). As post-translational modifications such as phosphorylation act as key modulators of LLPS (Li et al., 2022), we performed kinase enrichment analysis on proteins upregulated in the resistant cells and found that TANK binding kinase 1 (TBK1) ranked first among the elevated kinases (Fig. 4E). Coincidentally, GSK8612 as an inhibitor of p62 condensation and PC9 OsiR cell growth is a potent and selective TBK1 inhibitor. These data jointly prioritized GSK8612 and TBK1 as the focus of subsequent investigation.

### TBK1 phosphorylates p62 at Ser403 to drive condensation and EGFR-TKI resistance

To illustrate the role of TBK1 in p62 condensates after EGFR-TKI resistance, we first evaluated TBK1 expression levels in clinical specimens before and after resistance. Consistent with the p62 expression, the TBK1 level was remarkably enhanced post-resistance relative to pre-resistance (Fig. S6A and S6B). In PC9 OsiR cells, p62 pulled down TBK1, and reciprocally, TBK1 also pulled down p62 (Fig. S6C). Furthermore, more TBK1 co-precipitated with p62 in the resistant cells than in parental cells (Fig. S6D and S6E). Taken together, TBK1 may directly interact with p62 in EGFR-TKI-resistant lung cancer. To further determine whether TBK1 functionally contributes to EGFR-TKI resistance, TBK1 was knocked down in OsiR cells (Fig. S10A). TBK1 depletion markedly enhanced the sensitivity of OsiR cells to EGFR-TKI treatment (Fig. S7A and S7B). However, knockdown of interferon regulatory factor 3 (IRF3) or interferon regulatory factor 7 (IRF7) (Fig. S7C and S7D), two well-established downstream substrates of TBK1, had no obvious effect on EGFR-TKI sensitivity in OsiR cells (Fig. S7E–H). These results suggest that TBK1-mediated regulation of EGFR-TKI resistance is unlikely dependent on the canonical IRF3/IRF7 pathway.

We further evaluated p62 phosphorylation in parental and EGFR-TKI-resistant cells and observed a higher phosphorylation level at residues S349 and S403 of p62 in the resistant cells than that in the parental cells (Fig. S8A–F). These data were supported by a previous study regarding the phosphorylation of S349 and S403 in p62 condensates (Katsuragi et al., 2016; Matsumoto et al., 2011). Notably, the increase in phosphorylated p62 exceeded the total p62 upregulation (Fig. S8G–I). Furthermore, treatment with the TBK1 inhibitor GSK8612 induced a dose-dependent inhibition of p62 S403 phosphorylation, but not S349, in EGFR-TKI-resistant cells (Figs. 5A and S9A–D). Knockdown of TBK1 in OsiR cells significantly attenuated p62 phosphorylation at S403 (Fig. S10A–C). The immunofluorescence staining of p62 with S403 phosphorylated revealed that PC9 OsiR and HCC827 OsiR cells contained significantly larger condensate areas than their parental counterparts (Fig. S10D and S10E).

**Figure 5.**
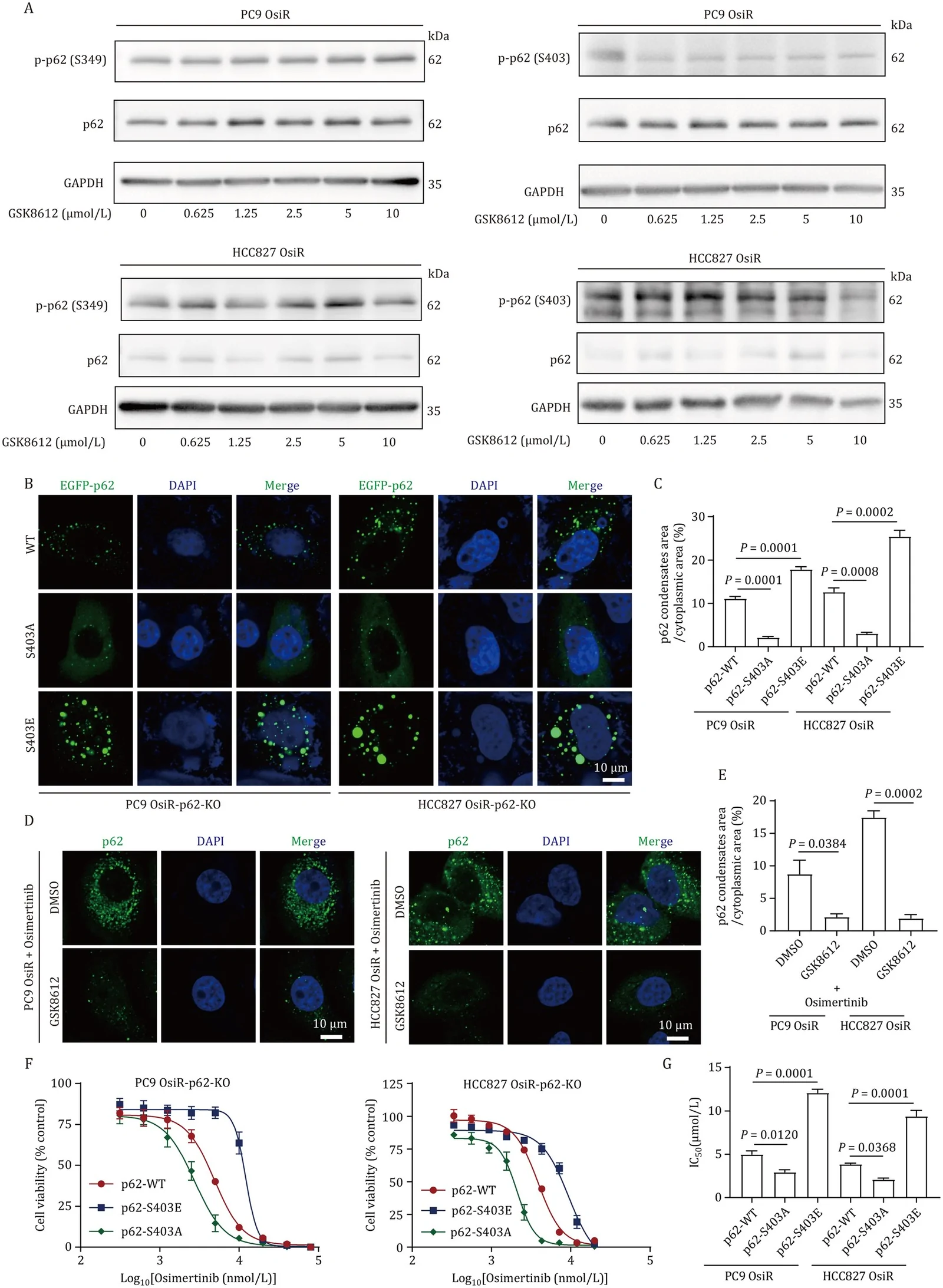
**TBK1 phosphorylates p62 at Ser403 to drive condensation and EGFR-TKI resistance**. (A) Western blot of phosphorylated p62 on residue S349 or S403 in OsiR cells following treatment of the TBK1 inhibitor GSK8612. (B) EGFP-p62 condensates in p62-KO OsiR cells rescued by wild-type, S403A, or S403E mutated p62. (C) The p62 condensates area as a percentage of cytoplasmic area (quantified across > 9 cells). *n *= 3 biological replicates. (D) Immunofluorescence of p62 condensates in OsiR cells treated with Osimertinib alone or combination with GSK8612. (E) The p62 condensates area as a percentage of cytoplasmic area (quantified across > 9 cells). *n *= 3 biological replicates. (F) CellTiter-Glo assay of cell viability in p62-KO OsiR cells rescued by either wild-type or point-mutated p62 (p62-S403E, p62-S403A), after 72 h treatment of Osimertinib. *n *= 3 biological replicates. (G) IC_50_ values of Osimertinib in OsiR cells. *n *= 3 biological replicates. Data are mean ± SEM; statistical analysis was performed using two-tailed Student’s *t*-test (E) and one-way ANOVA (C, G).

To determine the necessity of S403 phosphorylation, the WT, phospho-deficient p62-S403A, and phosphomimetic S403E mutant of p62 were ectopically expressed at matched protein levels in p62-KO cells (Fig. S10F). In comparison with p62-WT, p62-S403A reduced p62 condensate formation by 5.1-fold, 4.1-fold, and 4.0-fold in PC9 OsiR, HCC827 OsiR, and PC9 GefR cells, respectively, whereas p62-S403E enhanced p62 condensation by 1.6-fold, 2.0-fold, and 2.3-fold in these cells, respectively (Figs. 5B, 5C, S10G and S10H). To further evaluate the effect of S403 phosphorylation on p62 LLPS *in vitro*, we purified maltose-binding protein (MBP)-p62-WT-mCherry, MBP-p62-S403A-mCherry, and MBP-p62-S403E-mCherry proteins. Consistently, the phospho-deficient S403A mutation reduced the capacity of p62 droplet formation, while the phosphomimetic S403E mutation enhanced p62 droplet formation (Fig. S11A and S11B). The polyubiquitination mimicked by glutathione S-transferase (GST)-4×Ub promoted p62 droplet formation in a dose-dependent manner, and such a process was either reduced or promoted by S403A or S403E, respectively (Fig. S11C and S11D). These findings demonstrated that S403 phosphorylation directly regulates polyubiquitin chain induced p62 LLPS.

The pharmacological inhibition of TBK1 by GSK8612 in combination with EGFR-TKIs significantly reduced p62 condensate formation relative to EGFR-TKI treatment alone (Figs. 5D, 5E, S12A and S12B). Consistently, TBK1 knockdown substantially reduced p62 condensate formation in OsiR cells (Fig. S12C and S12D). Conversely, ectopic expression of TBK1 in parental cells markedly recovered p62 condensate formation (Fig. S12E–G). These findings further support a regulatory role of TBK1 in promoting p62 condensate assembly during EGFR-TKI resistance. Finally, we investigated whether phosphorylation of p62 at S403 functionally regulates EGFR-TKI sensitivity in resistant cells. In p62-KO resistant cells, reconstitution with the phosphomimetic p62 S403E mutant decreased sensitivity to EGFR-TKIs, leading to a more than 2-fold increase in IC_50_ values. Conversely, reconstitution with the phospho-deficient p62 S403A mutant enhanced EGFR-TKI sensitivity, reducing IC_50_ values by approximately 1.5-fold (Figs. 5F, 5G, S13A and S13B). However, in p62-KO parental cells (Fig. S13C), expression of either p62 S403E or p62 S403A had no obvious effect on EGFR-TKI sensitivity (Fig. S13D and S13E), suggesting that p62 S403 phosphorylation is required for maintaining EGFR-TKI resistance.

### GSK8612 reduces p62 condensation and restores sensitivity of EGFR-TKI-resistant lung cancer to EGFR-TKI treatment

Our findings nominate GSK8612 as a potential approach to overcome EGFR-TKI resistance through modulation of p62 condensation. To quantitatively evaluate the synergy between GSK8612 and EGFR-TKIs, we performed CellTiter-Glo viability assays in EGFR-TKI-resistant cells treated with various concentrations of EGFR-TKIs and GSK8612. Inclusion of GSK8612 markedly enhanced the sensitivity of resistant cells to EGFR-TKIs (Figs. 6A, S14A and S14B). The combination index (CI) calculated by CompuSyn was consistently below 1, indicating synergy between EGFR and TBK1 inhibition (Figs. 6B and S14C ). In addition, GSK8612 treatment failed to enhance EGFR-TKI sensitivity in p62-KO resistant cells (Fig. S14D and S14E), suggesting the p62-dependent function of TBK1 in EGFR-TKI resistance. In the absence of p62, GSK8612 failed to further enhance EGFR-TKI sensitivity in resistant cells, indicating that the ability of GSK8612 to restore EGFR-TKI responsiveness is largely p62-dependent. Although a low concentration of GSK8612 at 3 or 5 μmol/L did not suppress the growth of EGFR-TKI-resistant cells, the combination of GSK8612 with Osimertinib or Gefitinib markedly suppressed lung cancer cell proliferation in the colony formation assay (Figs. 6C, 6D, S14F and S14G). To further assess the *in vivo* effects of GSK8612, PC9 OsiR cells were subcutaneously implanted into nude mice to generate tumor xenografts. Mice were then randomized into four groups with treatment of Osimertinib (2.5 mg/kg/day, oral gavage) alone, GSK8612 (5 mg/kg/day, oral gavage) alone, combination of the two inhibitors, or the vehicle (Fig. 6E). Consistent with the cellular assay results, treatment with GSK8612 or Osimertinib alone had little to moderate suppression effect on tumor growth *in vivo*.

**Figure 6.**
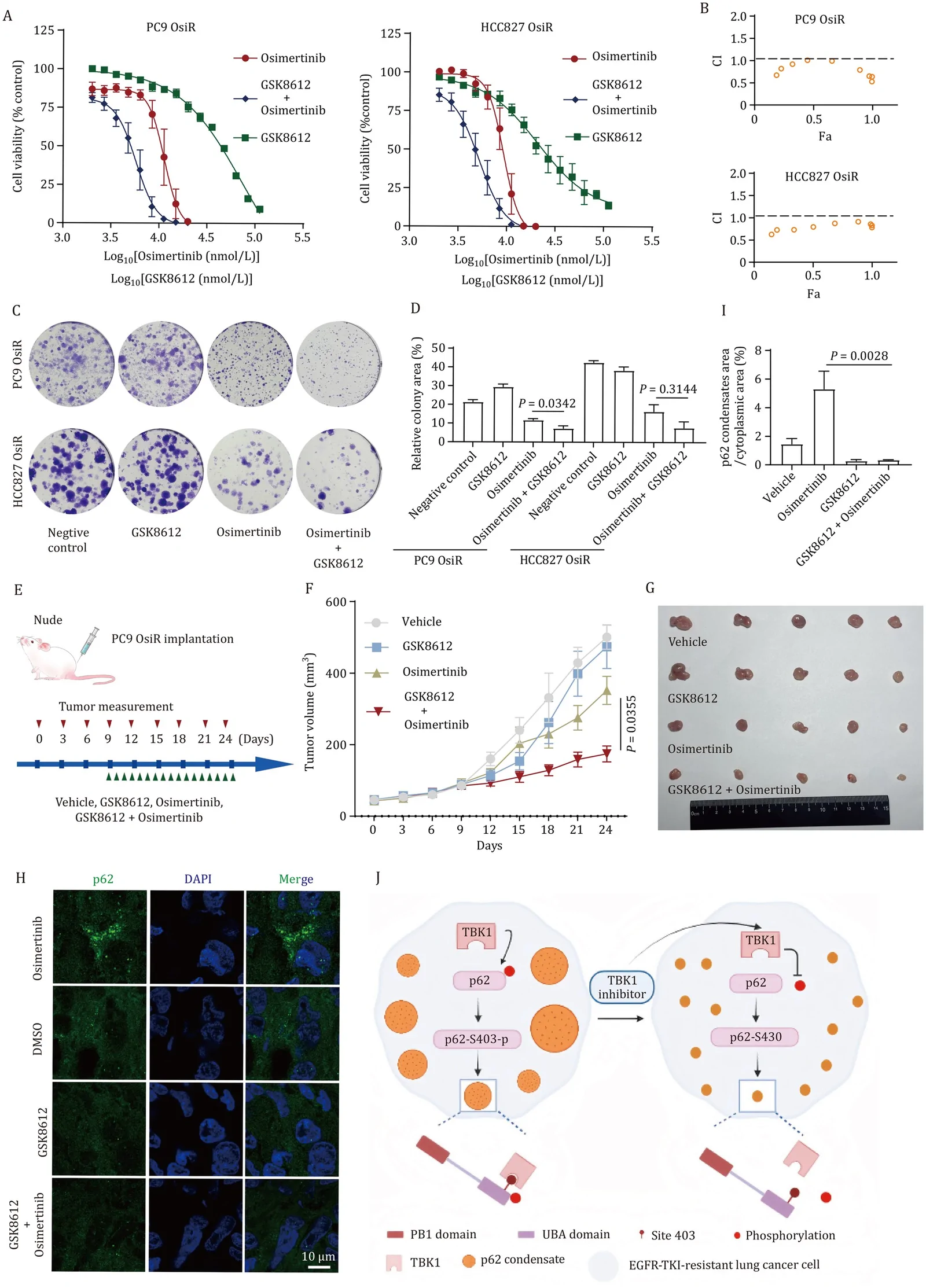
**GSK8612 reduces p62 condensation and restores sensitivity of EGFR-TKI-resistant lung cancer to EGFR-TKI treatment**. (A) CellTiter-Glo assay of cell viability in OsiR cells after 72 h treatment of Osimertinib alone, GSK8612 alone, or their combination at the indicated concentrations. *n *= 3 biological replicates. (B) CI values in PC9 OsiR and HCC827 OsiR cells were calculated using CompuSyn software based on CellTiter-Glo viability data and plotted as a function of fraction affected (Fa). (C) Colony formation assay of OsiR cells after treatment with Osimertinib, GSK8612 alone or in combination. *n *= 3 technical replicates. (D) Quantification of colony formation. *n *= 3 technical replicates. (E) PC9 OsiR cells (3.5 × 10^6^) were subcutaneously inoculated into nude mice for generating xenograft tumors. When the size of the tumors reached 100 mm^3^, the mice were randomly allocated into four groups and treated with the vehicle control (2% DMSO + 40% PEG300 + 5% Tween80 + 53% saline), Osimertinib (2.5 mg/kg/day, oral gavage), GSK8612 (5 mg/kg/day, oral gavage), or a combination of Osimertinib and GSK8612 for 15 days. *n *= 5 biological replicates. (F) The growth of tumor volumes measured on the indicated days. (G) At the end of the experiment, the tumors were dissected and imaged as indicated. (H) Immunofluorescence of p62 condensates in tumor tissue specimens from xenograft models. (I) Statistics of p62 condensate area in tumor tissue specimens from xenograft models (quantified across > 9 cells). *n *= 3 biological replicates. (J) Working model of targeting the TBK1-p62 condensate to restore sensitivity to EGFR-TKIs in resistant lung cancer, created by Biorender.com. Data are mean ± SEM; statistical analysis was performed using two-way ANOVA (D, F, I).

However, the combination of Osimertinib and GSK8612 remarkably inhibited tumor growth (Fig. 6F). Tumor volume in mice treated with Osimertinib monotherapy was approximately twice that observed with the combination regimen (Fig. 6G). None of these treatments induced statistically significant change in mouse body weight, indicating little toxicity associated with these treatments (Fig. S14H). Immunofluorescence staining of p62 on biopsies from mouse xenograft tumors demonstrated prominent p62 condensates in tumor tissues from mice treated with Osimertinib, whereas the treatment of GSK8612 either alone or in combination with Osimertinib almost completely suppressed the formation of p62 condensates (Fig. 6H and 6I). Taken together, our work outlines a therapeutic avenue for overcoming EGFR-TKI resistance in lung cancer through targeting p62 condensates.

## Discussion

Biomolecular condensates extend protein-centric views to a condensate-centric framework for cancer research, including tumor initiation, progression, therapy, and resistance (Mitrea et al., 2022). Leveraging clinical biopsies and *in vitro* and *in vivo* resistance models, this work proposes and validates a condensate-centric mechanism and intervention for EGFR-TKI resistance. Upon resistance to EGFR-TKIs, p62 undergoes LLPS-driven condensate amplification that sustains the resistant phenotype. TBK1-mediated phosphorylation of p62 at S403 emerges as the pivotal hub for condensate maintenance and drug tolerance. The selective TBK1 inhibitor GSK8612 reduces p62 S403 phosphorylation, destabilizes p62 condensates, and resensitizes cells to EGFR-TKI treatment. These findings directly link aberrant p62 phase separation, characterized by increased condensate formation and altered condensate dynamics in EGFR-TKI-resistant cells, to targeted-therapy resistance, suggesting that pharmacologic modulation of condensates is a tractable path to overcoming EGFR-TKI resistance (Fig. 6J). A few previous investigations have shown that EGFR-TKIs upregulate p62, thereby supporting tumor cell survival and reducing sensitivity to EGFR-TKIs (Yang et al., 2019). Activation of the YAP-p62 axis has also been reported in EGFR-TKI-resistant lung adenocarcinoma models (Park et al., 2021). However, a direct link between p62 condensates and EGFR-TKI resistance remains elusive. This study places the TBK1-p62-S403 axis in the context of resistance to EGFR inhibition, showing that EGFR blockade upregulates TBK1 and promotes its interaction with p62, selectively enhancing phosphorylation of p62 at S403 (but not S349) and thereby driving p62 condensate formation. The phosphomimetic S403E further enhances condensation and reduces sensitivity to EGFR-TKIs. These findings not only build on earlier findings that phosphorylation of p62 at S403 modulates the affinity of the UBA domain and LLPS (Sun et al., 2018), but also identify TBK1 as the responsible kinase in the context of EGFR-TKI resistance. This study offers greater disease-context specificity than the generalized understanding that TBK1 is activated at p62 condensates (Schlütermann et al., 2021). In resistant cells, p62 condensates fuse more slowly than in parental cells, suggesting distinct properties in phase separation and droplet dynamics between the two states. This may be attributed to the long-term influence of the drug, which results in more significant structural changes in intracellular condensates and phase separation. The condensation-prone p62-S403E mutagenesis promotes EGFR-TKI resistance, while the condensation-inhibitory p62-S403A mutagensis enhances the sensitivity of EGFR-TKI treatment, thus supporting the causality between p62 condensation and TKI resistance. However, because S403 is located within the UBA domain, which is the core domain responsible for recognizing ubiquitinated substrates and serves as the cargo-recognition module of p62, it remains challenging to fully disentangle the intrinsic cargo-binding function of p62 from its condensate-associated function (Sun et al., 2018; You et al., 2019). It remains challenging to fully disentangle the intrinsic functions of a protein from its condensate-associated functions (Alberti et al., 2019; Holehouse and Alberti, 2025).

Previous research from an innate-immunity perspective has revealed that EGFR inhibition triggers the type I interferon-signaling pathway mediated by the RIG-I-TBK1-IRF3 axis, thereby conferring resistance to EGFR-TKIs, whereas inhibiting interferon signaling restores sensitivity (Gong et al., 2020). Building on this, the present study advances understanding of EGFR-TKI resistance mechanisms from the signaling-pathway level to biomolecular phase separation, showing that TBK1-p62 condensate phosphorylation regulates EGFR-TKI resistance and proposing a framework linking dysregulation of condensate homeostasis to targeted-therapy resistance. Prior studies indicate that the selective TBK1 inhibitor GSK8612 exerts anti-tumor activity across multiple models, e.g., suppression of hepatocellular carcinoma cell growth with increased CD8^+^ T-cell infiltration, blockade of the TBK1-p62 axis to curb cholangiocarcinoma cell proliferation and metastasis, and enhancement of anthracycline sensitivity in leukemia cells via the AKT-CDK2 pathway (Chen et al., 2021; Gao et al., 2023; Jiang et al., 2021). In this study, we screened our in-house compound library for modulators of p62 condensates and identified GSK8612 along with 11 additional compounds that either suppress or promote p62 droplet formation in EGFR-TKI-resistant lung cancer cells. As post-translational modifications are efficacious modulators of LLPS (Qin et al., 2024; Zhang et al., 2024), here we found TBK1 was the most elevated kinase in resistant versus parental cells. The coincidence of these observations suggests an essential role of TBK1-p62 in droplet formation and drug resistance. GSK8612 suppressed p62 droplet formation likely through the inhibition of p62 S403 phosphorylation, and eventually resensitized EGFR-TKI treatment, both in cell lines and in xenografts. The low dose of GSK8612 (a TBK1 inhibitor) does not induce nonspecific cytotoxicity but rather relieves a resistance-specific ‘buffer’ to EGFR-TKI-induced stress, thereby revealing the dependence of resistant cells on p62 condensate homeostasis. It presents a low-toxicity, LLPS-driven combination strategy for therapeutic indications of GSK8612. We propose that, when EGFR signaling is suppressed, the TBK1-p62 axis promotes condensate formation in tumor cells to mediate proteotoxic and autophagy/proteostasis stress, towards a sustained cell viability and proliferation.

In conclusion, this study uncovers a condensate-centric mechanism of EGFR-TKI resistance in lung cancer, wherein TBK1-regulated phosphorylation of p62 S403 sustains cytoplasmic p62 condensates and facilitates the acquisition of resistance in EGFR-mutant tumors. On the basis of these findings, we propose that inhibition of the TBK1-p62 axis by the TBK1 inhibitor GSK8612 constitutes a condensate-targeted therapeutic strategy to enhance the sensitivity of EGFR-TKI treatment in EGFR-TKI resistant lung cancer.

## Supplementary Material

pwag043_Supplementary_Data

## Data Availability

The mass spectrometry proteomics data from clinical biopsy specimens have been deposited to the ProteomeXchange Consortium via the iProX partner repository with the dataset identifier PXD070390. The cell-derived proteomic dataset analyzed in this study was obtained from the publicly available Synapse database under accession ID syn25915977.

## Code availability

No custom code or software was generated in this study.

## Artificial intelligence (AI)

During the preparation of this manuscript, the authors used AI-assisted tools only for language editing and grammar improvement.

## Abbreviations

Baf A1: bafilomycin A1
CI: combination index
CTSD: cathepsin D
DMSO: dimethyl sulfoxide
EGFP: enhanced green fluorescent protein
EGFR: epidermal growth factor receptor
EGFR-TKI: epidermal growth factor receptor tyrosine kinase inhibitor
FRAP: fluorescence recovery after photobleaching
GefR: gefitinib-resistant
GST: glutathione S-transferase
IC_50_: half-maximal inhibitory concentration
IRF3: interferon regulatory factor 3
IRF7: interferon regulatory factor 7
KO: knockout
LC3: microtubule-associated protein 1 light chain 3
LLPS: liquid-liquid phase separation
MBP: maltose-binding protein
NSCLC: non-small cell lung cancer
OsiR: osimertinib-resistant
PB1: , Phox and Bem1p domain
PONDR: predictor of natural disordered regions
RI: resistance index
SQSTM1/p62: sequestosome 1/p62
TBK1: TANK-binding kinase 1
UBA: ubiquitin-associated domain
WT: wild type
