# Supplementary material for: Targeting the TBK1-p62 condensate axis restores sensitivity to EGFR-TKIs in resistant lung cancer
Source: Protein Cell. 2026 Jun 26;17(11):1021–34. doi: 10.1093/procel/pwag043 (PMC13721109; doi:10.1093/procel/pwag043)
Supplement: Supplementary file 1 [file pwag043_supplementary_data.zip › 2 Supplementary information.docx]

**Materials and Methods**

**Cell culture and drug-resistant model establishment**

The human NSCLC cell lines PC9 and HCC827, both carrying EGFR exon 19 deletions, were purchased from the Cell Bank of the Chinese Academy of Sciences. HEK293T cells were purchased from ZQXZBIO. To generate Osimertinib-resistant cell lines, parental cells were continuously exposed to stepwise increasing concentrations of Osimertinib (MedchemExpress, HY-15772) for more than 6 months, following a previously described protocol (Nishihara et al., 2022; Tang et al., 2016). PC9 OsiR and HCC827 OsiR cells were maintained in 1 μM and 0.5 μM Osimertinib, respectively, to preserve their resistant phenotypes. The Gefitinib resistant PC9 GefR cell line (resistance index >10) was kindly provided by the research group of Professor Yueyin Pan from the Department of Oncology, The First Affiliated Hospital of University of Science and Technology of China. PC9 GefR cells were maintained in 6 μM Gefitinib (MedchemExpress, HY-50895) to preserve their resistant phenotype. PC9, HCC827, PC9 OsiR, HCC827 OsiR, PC9 GefR and HEK293T cells were maintained in RPMI-1640 medium (Gibco, 6125138) or DMEM medium (Gibco, 6123106) supplemented with 10% fetal bovine serum (Gibco, 2800811), 100 U mL^−1^ penicillin and 100 μg mL^−1^ streptomycin (Beyotime Biotechnology, C0222). All the cells were cultured at 37 °C under 5% CO_2_ and were free of mycoplasma contamination.

**Patients and specimens**

Ethical approval for this study was granted by the Ethics Committee of the First Affiliated Hospital of Anhui Medical University (PJ2024-11-59), and all participants provided written informed consent. Three EGFR-TKI-naïve and EGFR-TKI-resistant needle biopsy specimens were obtained from six patients with EGFR-mutant NSCLC diagnosed at the First Affiliated Hospital of Anhui Medical University between 2024 and 2025 (Supplementary Table 1).

**Plasmids, siRNA and transient transfection**

The p62-WT, p62-ΔPB1, and p62-ΔUBA genes were chemically synthesized by Sangon Biotech and cloned into the pEGFP-C3 (Sangon Biotech), pcDNA3.1(+)-C-DYK (Sangon Biotech), and pCMV-C-BioID2-Flag (Beyotime Biotechnology, D3023) vectors using ready-to-use seamless cloning kitstandard cloning kits (Sangon Biotech, B632219). p62-S403E was generated by site-directed mutagenesis kit (Sangon Biotech, B639281) and confirmed by DNA sequencing. Negative control (5′-UUCUCCGAACGUGUCACGUTT-3′), p62-siRNA-1 (5′-CAUGUCCUACGUGAAGGAUGA-3′) and p62-siRNA-2 (5′-GCAUUGAAGUUGAUAUCGAU-3′) were directly synthesized by Sangon Biotech. For plasmid transfections, cells were seeded overnight, and plasmids were transfected with Lipofectamine™ 3000 transfection reagent (Thermo Fisher Scientific, L3000015) according to the manufacturer’s instructions. For siRNA transfections, cells were seeded overnight and transfected with Lipofectamine™ 2000 transfection reagent (Thermo Fisher Scientific, 11668019) according to the manufacturer’s instructions. Follow-up experiments were conducted 48 h post-transfection.

**Lentiviral transduction and the generation of stable cell lines**

CRISPR-Cas9 genomic editing for gene deletion, the gRNAs targeting p62 were designed as follows: p62-gRNA-1: 5′-AAUGGCCAUGUCCUACGUGA-3′; p62-gRNA-2: 5′-UUCACACUCGGAUCCUCCGA-3′. The gRNAs were cloned into the LentiCRISPR v2 vector (GenScript) for lentiviral delivery. The HEK293T cells were transfected with the expression plasmid along with the packaging plasmids pMD2.G (MiaoLing plasmid platform, P0262) and pSPAX2 (MiaoLing plasmid platform, P0261) using Lipofectamine™ 2000 transfection reagent. The cell culture supernatant collected at 24 h was discarded, while the supernatant harvested at 72 h was collected and used for infecting cells or stored at −80 °C. To generate stable cell lines, the cells were infected for 24 h in a mixture consisting of a 1:1 ratio of normal culture medium to lentiviral supernatant, supplemented with polybrene (Hanbio, HB-PB-500) at a final concentration of 1 µg mL^−1^. After 48 h, the cells were continuously selected with 2 µg mL^−1^ puromycin (Beyotime Biotechnology, ST551) for three passages, and the resulting derivative cells were maintained in 0.5 µg mL^−1^ puromycin.

**Ultra-low-input quantitative proteomics analysis**

**Protein extraction and digestion**

Protein samples were digested in-solution according to the following procedure. Samples were first incubated with 20 mM detergent for 2 h at 37 °C. After cooling, 40 mM alkylating agent was added, gently mixed, and incubated in the dark for 30 min. Sequential addition of Tris (2-carboxyethyl) phosphine (3 μL) and Iodoacetamide (5 μL) was performed. The alkylation reaction was terminated by heating at 95 °C, followed by tryptic digestion for 2 h at 37 °C. The digested peptides were desalted, vacuum-concentrated, and reconstituted in 0.1% formic acid. Prior to DIA, indexed retention time peptides were spiked into each sample (400 ng/sample).

**LC-MS/MS Analysis**

Peptide samples were analyzed on an Orbitrap™ Astral™ mass spectrometer (Thermo Scientific) coupled to a Vanquish Neo liquid chromatography system (Thermo Scientific) operated in data-independent acquisition (DIA) mode. Precursor ions were scanned over an m/z range of 380–980 with an MS1 resolution of 240,000 at 200 m/z, a normalized AGC target of 500%, and a maximum injection time of 5 ms. For DIA MS2 acquisition, 299 windows were used with an isolation window of 2 m/z, higher-energy collisional dissociation (HCD) collision energy of 25 eV, a normalized AGC target of 500%, and a maximum injection time of 3 ms.

**Mass spectrometry data analysis**

DIA data were processed using DIA-NN (version 1.8.1) with the following settings: enzyme, trypsin (maximum of one missed cleavage); fixed modification, carbamidomethylation of cysteine (C); variable modifications, methionine oxidation (M) and N-terminal acetylation. All reported protein identifications were filtered at a 1% false discovery rate (FDR) to ensure 99% confidence. The mass spectrometry proteomics data have been deposited to the ProteomeXchange Consortium via the iProX partner repository (Chen et al., 2022; Ma et al., 2019) with the dataset identifier PXD070390.

**Immunofluorescence**

Cells were seeded in chambered cell culture slide (LABSELECT, CS-08-TC), followed by plasmid transfection or drug treatment. Cells or frozen sections were fixed with 4% paraformaldehyde (Beyotime Biotechnology, P0099) for 30 min at room temperature. Permeabilized with immunostaining permeabilization buffer with saponin (Beyotime Biotechnology, P0095) for 15 min. Blocked with 3% bovine serum albumin (BSA) (Biosharp, BS114) for 1 h. Incubated with primary antibody against p62 (Proteintech, 66184-1-Ig, 1:400) or against p-p62 (S403) (GeneTex, GTX128171, 1:500) overnight at 4 °C, followed by incubation with fluorophore-conjugated secondary antibody (Beyotime Biotechnology, A0423, 1:500 or A0216, 1:500) for 1 h at room temperature. Stained nuclei with DAPI. Images were acquired using a Zeiss LSM 980 confocal microscope (100× oil objective). Quantification of condensate area relative to cytoplasmic area was performed using ImageJ.

**Cellular FRAP assays**

Cells were transfected with p62-EGFP plasmid and maintained at 37 °C in a live-cell imaging chamber. FRAP imaging was carried out with a Zeiss LSM 980 confocal microscope.A defined region of interest (ROI) was photobleached with a 488-nm laser at 50% power for 2 s. Time-lapse images were acquired for 200 s post-bleaching at 2s intervals. Images were processed using ImageJ to quantify fluorescence recovery.

**Cell viability assay**

Cells were planted into 96-well plates and then incubated with Osimertinib, Gefitinib or compounds for 72 h. Cell viability was detected by CellTiter-Glo Luminescent Cell Viability (Promega, G7570), following the manufacturer’s instructions. Luminescence signals were measured using the chemiluminescence detection module of a Molecular Devices Spectramax iD5 microplate reader.

**Western blot**

Cell lysis was performed on ice using RIPA buffer (Beyotime Biotechnology, P0013B), and supernatants were obtained by centrifugation. Protein levels were quantified with a BCA protein assay kit (Beyotime Biotechnology, P0009). Proteins were separated by 10% SDS-PAGE and transferred onto PVDF membranes. The membranes were blocked with protein-free rapid blocking buffer (Epizyme Biotech, PS108P) for 15 min at room temperature, followed by incubation with primary antibodies overnight at 4 °C and HRP-conjugated secondary antibodies for 1 h at room temperature. Bound antibodies were visualized by ECL reagents (Beyotime Biotechnology, P0018S). Band intensities were quantified using ImageJ software. Antibodies: p62 (Proteintech, 66184-1-Ig, 1:2000); p-p62 S403 (GeneTex, GTX128171, 1:2000); p-p62 S349 (Proteintech, 29503-1-AP, 1:6000); TBK1 (Proteintech, 28397-1-AP, 1:2000); LC3 (Proteintech, 14600-1-AP, 1:2000); CTSD (Wanleibio, WL01234, 1:2000); IRF3 (Wanleibio, WL02431, 1:2000); IRF7 (Proteintech, 22392-1-AP, 1:2000); Vinculin (Proteintech, 26520-1-AP, 1:5000); GAPDH (Beyotime Biotechnology, AF0006, 1:2000).

**Immunohistochemistry**

Tissue sections were deparaffinized in xylene and rehydrated through a graded ethanol series. Antigen retrieval was performed by microwaving the slides in citrate buffer (pH 6.0), followed by quenching of endogenous peroxidase activity with 0.3% hydrogen peroxide. After blocking with 3% BSA (Biosharp, BS114), slides were incubated with primary antibodies overnight at 4 °C in the dark. The slides were incubated with an HRP-conjugated secondary antibody for 2 h at room temperature, followed by color development using a DAB substrate kit (Servicebio, G1212). Tissue sections for IHC were scanned employing the TissueGnostics Panorama Tissue & Cell Quantitative Analysis System (63× objective). IHC staining was quantified using the Image-Pro Plus software. Antibodies: p62 (Proteintech, 66184-1-Ig, 1:2000); TBK1 (Proteintech, 28397-1-AP, 1:200).

**Drug screening**

Cells were seeded in chambered cell culture slide (LABSELECT, CS-08-TC), followed by different compound treatment. Primary Screening: cells were treated with compound mixtures (50 μM each) from our in-house compound library for 3 h. Positive control: 10% 1,6-hexanediol (phase separation disruptor). Negative control: DMSO. Following mixtures treatment, the samples were subjected to Immunofluorescence staining for p62 condensates. Imaging was performed utilizing the TissueGnostics Panorama Tissue & Cell Quantitative Analysis System (63× oil objective). Quantification of condensate area relative to cytoplasmic area was performed using ImageJ. Secondary Screening: hit compounds from the primary screen were retested as single agents (50 μM, 3 h). All subsequent steps were identical to those described above.

**BioID2-coupled Mass spectrometry**

HCC827 and HCC827 OsiR cells were separately seeded into 10 cm dishes. On the following day, the cells were transfected with the p62-WT-BioID2 plasmid. After 3 h, biotin was added to the culture medium at a final concentration of 3.6 µM. After 24 h, cells were lysed using Western and IP lysis buffer (Beyotime Biotechnology, P0013), and the lysates were centrifuged to collect the supernatants. The supernatant was then incubated with streptavidin magnetic beads (Beyotime Biotechnology, P2151) overnight at 4 °C. Subsequently, the bound proteins were eluted with an acidic elution buffer (0.1 M Glycine, pH 2.0). The eluate was separated from the beads using a magnetic rack and collected for further analysis. Identification of interacting and proximal Proteins by Mass Spectrometry.

**Co-immunoprecipitation (Co-IP)**

Cells were harvested, lysed in IP buffer, and centrifuged to collect the supernatant. Protein G magnetic beads (Beyotime Biotechnology, P2105) were resuspended in 500 μL of either the specific antibody working solution or normal IgG working solution and incubated with rotation at room temperature for 1 h. The cell lysate was first pre-cleared by incubation with the IgG-conjugated beads at 4 °C for 1 h, followed by magnetic separation. The resulting supernatant was then subjected to immunoprecipitation by incubation with the antibody-conjugated beads on a rotary mixer overnight at 4 °C. After magnetic separation and washing, the bound complexes were eluted with 1× SDS-PAGE loading buffer (Beyotime Biotechnology, P0015A). The final eluate was collected via magnetic separation for subsequent Western blot analysis. Antibodies: p62 (Proteintech, 66184-1-Ig); TBK1 (Proteintech, 28397-1-AP). IP: 4 μg antibody for 3 mg of total protein lysate.

**Colony formation assay**

Clonogenicity was assessed by plating 1,000 cells (PC9 OsiR, HCC827 OsiR, or PC9 GefR) per well in six-well plates. The next day, cells were exposed to the indicated drugs, and the culture medium was changed every 3–4 days thereafter. After culturing for 10–14 days, colonies were fixed with 4% paraformaldehyde (Beyotime Biotechnology, P0099) for 15 min and then stained with crystal violet (Beyotime Biotechnology, C0121) for 30 min. Quantification was performed by photographic documentation and statistical analysis of the colony area.

**Animal experiments**

All mice experiments were approved by the Experimental Animal Welfare and Ethics Committee of Hefei Comprehensive National Health Science Center and conducted in accordance with Institutional Animal Care and Use Committee guidelines (IHM-AP-2025-018).

In order to assess the effect of p62 on Osimertinib sensitivity, 3.5 × 10⁶ PC9 OsiR or PC9 OsiR-p62-KO cells were subcutaneously implanted into immunodeficient mice for generating xenograft tumors (*n* = 3, per group). Nude mice were purchased from Shanghai Model Organisms Center. When tumors reached 100 mm³, mice were randomized into: Vehicle control: 2% DMSO + 40% PEG300 + 5% Tween80 + 53% saline or Osimertinib: 2.5 mg/kg/day (oral gavage). Treatments were administered for 19 days. The length (L) and width (W) of the tumors were measured by a caliper at the indicated time, and the volume of the tumors was calculated using the formular: (L × W^2^) /2.

In order to verify the effect of GSK8612 on Osimertinib resistance, 3.5×10⁶ PC9 OsiR cells were subcutaneously implanted into nude mice for generating xenograft tumors (*n* = 5, per group). When tumors reached 100 mm³, mice were randomized into: Vehicle control: 2% DMSO + 40% PEG300 + 5% Tween80 + 53% saline, Osimertinib: (2.5 mg/kg/day, oral gavage), GSK8612: (5 mg/kg/day, oral gavage) or a combination of Osimertinib and GSK8612. Treatments were administered for 15 days. The length (L) and width (W) of the tumors were measured by a caliper at the indicated time, and the volume of the tumors was calculated using the formular: (L × W^2)^ /2. At the experimental endpoint, tumors were harvested and processed for Immunofluorescence analysis.

***In vitro* phase separation assay of p62 condensate**

Recombinant MBP-p62-WT-mCherry, MBP-p62-S403A-mCherry, MBP-p62-S403E- mCherry, and GST-4×Ub proteins were expressed in Escherichia coli BL21 Rosetta (DE3) cells. Bacterial pellets were resuspended in lysis buffer containing 1 M NaCl, 40 mM Tris-HCl (pH 7.5), 10% glycerol, 0.05% Triton X-100, 1 mM TCEP, and protease inhibitor cocktail. After sonication and centrifugation, MBP-tagged p62 proteins were purified using an MBPSep dextrin agarose resin, whereas GST-4×Ub was purified by glutathione affinity chromatography. After affinity purification, the proteins were further purified by Superdex 200 size-exclusion chromatography, followed by buffer exchange into 150 mM NaCl, 40 mM Tris-HCl (pH 7.5), 10% glycerol, 1 mM TCEP. The peak fractions were collected for subsequent *in vitro* phase separation assays.

For the *in vitro* phase separation assay, purified p62 proteins were mixed with GST-4×Ub at the indicated final concentrations in phase separation buffer and incubated at room temperature for 10 min. For concentration-gradient experiments, p62 proteins or GST-4×Ub were added at the indicated concentrations while keeping the other component constant. The reaction mixtures were then transferred to glass-bottom dishes and imaged using a confocal fluorescence microscope.

**Statistical analysis**

All analyses were performed under blinded conditions with respect to the experimental groups. Unless otherwise stated, quantitative data are expressed as the mean ± s.e.m. from at least three independent biological replicates or. technical replicates. Differences between groups were evaluated for statistical significance using two-tailed Student's t-test, one-way ANOVA, or two-way ANOVA, as appropriate, with a p value of less than 0.05 considered statistically significant (*P* < 0.05). Detailed statistical parameters for all experiments are included in the corresponding figure legends to ensure reproducibility. Throughout this study, samples were randomly assigned to the experimental groups. Data from all Western blot and cell culture-based assays are representative of at least three independent biological replicates. For *in vitro* studies, sample sizes consisted of a minimum of three replicates. For *in vivo* experiments, group sizes ranged from 3 to 5 animals.

**References**

Nishihara S, Yamaoka T, Ishikawa F et al. Diverse mechanisms of resistance against osimertinib, a third-generation EGFR-TKI, in lung adenocarcinoma cells with an EGFR-activating mutation. Cells 2022;**11**:2201.

Tang Z-H, Jiang X-M, Guo X et al. Characterization of osimertinib (AZD9291)-resistant non-small cell lung cancer NCI-H1975/OSIR cell line. Oncotarget 2016;**7**:81598–81610.

Ma J, Chen T, Wu S et al. iProX: an integrated proteome resource. Nucleic Acids Res 2019;**47**:D1211–D1217.

Chen T, Ma J, Liu Y et al. iProX in 2021: connecting proteomics data sharing with big data. Nucleic Acids Res 2022;**50**:D1522–D1527.

**Supplemental figure legends**

**Fig. S1 Establishment of EGFR-TKI-resistant lung cancer cell lines.** (A) The cell viability of PC9 and PC9 OsiR cells assessed by CellTiter-Glo after 72 h treatment of Osimertinib. *n* = 3 biological replicates. (B) The cell viability of HCC827 and HCC827 OsiR cells assessed by CellTiter-Glo after 72 h treatment of Osimertinib. *n* = 3 biological replicates. (C) The cell viability of PC9 and PC9 GefR cells assessed by CellTiter-Glo after 72 h treatment of Gefitinib. *n* = 3 biological replicates. (D) EGFR mRNA sequencing comparison between PC9 and PC9 OsiR cells. Data are mean ± s.e.m..

**Fig. S2 p62 condensates contribute to Gefitinib resistance in PC9 GefR cells.** (A) Immunofluorescence of p62 condensates in PC9 and GefR cells. (B) The p62 condensates area as a percentage of the cytoplasmic area (quantified across > 9 cells). *n* = 3 biological replicates. (C) Western blot of p62-KO in OsiR and GefR cells. (D) EGFP-p62 condensates in p62-KO GefR cells rescued by wild-type or PB1/UBA domain deleted p62. (E) The p62 condensates area as a percentage of cytoplasmic area (quantified across > 9 cells). *n* = 3 biological replicates. (F) CellTiter-Glo assay of cell viability in p62-KO GefR cells rescued by either wild-type or domain-deleted p62, after 72 h treatment of Gefitinib. *n* = 3 biological replicates. (G) IC_50_ values of Gefitinib in GefR cells. *n* = 3 biological replicates. Data are mean ± s.e.m.; statistical analysis was performed using two-tailed Student’s *t*-test (B) or one-way ANOVA (G).

**Fig. S3 The p62 protein is a key mediator of Gefitinib resistance in PC9 GefR cells.** (A) Western blot of p62 in parental or Gefitinib resistant cells. (B) The statistics of the p62 protein expression level. *n* = 3 biological replicates. (C) The growth curves of OsiR and GefR cells were determined using the CellTiter-Glo assay. (D) CellTiter-Glo assay of cell viability in p62-KO GefR cells after 120 h treatment of Gefitinib. n = 3 biological replicates. (E) IC_50_ values of Gefitinib in GefR cells. *n* = 3 biological replicates. (F) Representative images of plate colony formation assay. OsiR, OsiR-p62-KO, GefR and GefR-p62-KO cells were seeded at the same density, then treated with DMSO control or EGFR-TKIs continuously. After culturing until visible colonies formed, colony growth was visualized by crystal violet staining. (G) Quantitative analysis of colony formation comparing OsiR, OsiR-p62-KO, GefR and GefR-p62-KO cells, with total colony area calculated for each group. (H) Western blot of p62 overexpression in OsiR and GefR cells. (I) CellTiter-Glo assay of cell viability in p62 overexpression OsiR cells after 72 h treatment of Gefitinib. *n* = 3 biological replicates. (J) IC_50_ values of Gefitinib in GefR cells. *n* = 3 biological replicates. Data are mean ± s.e.m.; statistical analysis was performed using two-tailed Student’s *t*-test (B, E, J) or two-way ANOVA (G).

**Fig. S4 EGFR-TKI-resistant cells exhibit enhanced autophagic flux and lysosomal activation.** (A) Western blot analysis of p62 in parental and OsiR cells treated with or without BafA1. (B) Quantification of relative Δp62 levels. Δp62 = p62(+Baf) − p62(−Baf). n = 3 biological replicates. (C) Western blot analysis of LC3-Ⅰ, LC3-Ⅱ in parental and OsiR cells treated with or without BafA1. (D) Quantification of relative LC3-II turnover. ΔLC3-II = LC3-II(+Baf) − LC3-II(−Baf). n = 3 biological replicates. (E) Representative images of LysoSensor and LysoTracker staining in parental and OsiR cells. (F) Quantification of LysoSensor and LysoTracker fluorescence density (quantified across > 9 cells). n = 3 biological replicates. (G) Western blot analysis of pro-CTSD, intermediate CTSD and mature CTSD in parental and OsiR cells, with vinculin as the loading control. (H) Quantification of pro-CTSD and mature CTSD expression normalized to vinculin. n = 3 biological replicates. (I) Quantification of the mature CTSD / pro-CTSD ratio. n = 3 biological replicates. Data are mean ± s.e.m.; statistical analysis was performed using two-tailed Student’s t-test (B, D, F, H, I).

**Fig. S5 Compounds that inhibit or promote p62 condensate formation.** (A) The effects of different cocktails (50 μM, 3 h) on the area of p62 condensates in PC9 OsiR cells. n.s., not significant (quantified across > 3 cells). n=3 technical replicates. (B) Immunofluorescence analysis of p62 condensates in PC9 OsiR cells treated with in-house library compounds that inhibit p62 condensates formation. *n*=3 technical replicates. (C) Immunofluorescence analysis of p62 condensates in PC9 OsiR cells treated with in-house library compounds that promote p62 condensates formation. *n*=3 technical replicates. Data are mean ± s.e.m.; statistical analysis was performed using two-tailed Student’s *t*-test (A).

**Fig. S6 Increased TBK1-p62 interaction accompanies EGFR-TKI resistance.** (A) The TBK1 IHC in lung cancer biopsies obtained before treatment and after the development of EGFR-TKI resistance. (B) Statistics of the TBK1 expression level in TKI-naïve or resistant lung cancer biopsies. *n* = 3 biological replicates. (C) Western blot of p62-TBK1 reciprocal CO-IP in PC9 OsiR cells: p62 pulldown probing TBK1 (left) and TBK1 pulldown probing p62 (right). (D) Western blot of p62 pulldown probing TBK1 in PC9 and PC9 OsiR cells. (E) The statistics of the TBK1/p62 protein expression level. *n* = 3 biological replicates. Data are mean ± s.e.m.; statistical analysis was performed using two-tailed Student’s *t*-test (B, E).

**Fig. S7 TBK1 knockdown sensitizes EGFR-TKI-resistant cells to EGFR-TKIs independently of IRF3/IRF7.** (A) CellTiter-Glo assay of cell viability in TBK1 knockdown OsiR cells after 72 h treatment of Osimertinib. n = 3 biological replicates. (B) IC_50_ values of Osimertinib in OsiR cells. n = 3 biological replicates. (C) Western blot of IRF3 knockdown in OsiR cells. (D) Western blot of IRF7 knockdown in OsiR cells. (E) CellTiter-Glo assay of cell viability in IRF3 knockdown OsiR cells after 72 h treatment of Osimertinib. n = 3 biological replicates. (F) IC_50_ values of Osimertinib in OsiR cells. n = 3 biological replicates. (G) CellTiter-Glo assay of cell viability in IRF7 knockdown OsiR cells after 72 h treatment of Osimertinib. n = 3 biological replicates. (H) IC_50_ values of Osimertinib in OsiR cells. n = 3 biological replicates. Data are mean ± s.e.m.; statistical analysis was performed using one-way ANOVA (B, F, H).

**Fig. S8 p62 S349/S403 phosphorylation is elevated in EGFR-TKI-resistant-cells.** (A) Western blot of phosphorylation levels at S349 (p-p62 S349) (left) and S403 (p-p62 S403) (right) in PC9 OsiR cells. (B) The statistics of the p-p62 (S349)/GAPDH and p-p62 (S403)/GAPDH protein expression level. *n* = 3 biological replicates. (C) Western blot of phosphorylation levels at S349 (p-p62 S349) (left) and S403 (p-p62 S403) (right) in HCC827 OsiR cells. (D) The statistics of the p-p62 (S349)/GAPDH and p-p62 (S403)/GAPDH protein expression level. *n* = 3 biological replicates. (E) Western blot of phosphorylation levels at S349 (p-p62 S349) (left) and S403 (p-p62 S403) (right) in PC9 GefR cells. (F) The statistics of the p-p62 (S349)/GAPDH and p-p62 (S403)/GAPDH protein expression level. *n* = 3 biological replicates. G–I, The statistics of the p-p62 (S349)/p62 and p-p62 (S403)/p62 protein expression level. *n* = 3 biological replicates. Data are mean ± s.e.m.; statistical analysis was performed using two-tailed Student’s *t*-test (B, D, F–I).

**Fig. S9 GSK8612 dose-dependently reduces p62 S403 phosphorylation while sparing S349.** (A) The statistics of the of p-p62 (S349)/p62 (left) and p-p62 (S403)/p62 (right) protein expression level in PC9 OsiR cells following treatment with increasing concentrations of the TBK1 inhibitor GSK8612. *n* = 3 biological replicates. (B) The statistics of the p-p62 (S349)/p62 (left) and p-p62 (S403)/p62 (right) protein expression level in HCC827 OsiR cells following treatment with increasing concentrations of the TBK1 inhibitor GSK8612. *n* = 3 biological replicates. (C) Western blot of p-p62 S349 (left) and p-p62 S403 (right) in PC9 GefR cells following treatment with increasing concentrations of the TBK1 inhibitor GSK8612. (D) The statistics of the p-p62 (S349)/p62 (left) and p-p62 (S403)/p62 (right) protein expression level in PC9 GefR cells following treatment with increasing concentrations of the TBK1 inhibitor GSK8612. *n* = 3 biological replicates. Data are mean ± s.e.m.; statistical analysis was performed using one-way ANOVA (A, B, D).

**Fig. S10 TBK1-dependent p62 phosphorylation drives p62 condensates formation in EGFR-TKI-resistant cells.** (A) Western blot of TBK1 and p-p62 S403 in OsiR cells transfected with negative control siRNA or two independent TBK1-targeting siRNAs. (B) The statistics of the TBK1/GAPDH protein expression level in PC9 OsiR cells (left) and HCC827 OsiR cells (right). *n* = 3 biological replicates. (C) The statistics of the p-p62 (S403)/GAPDH protein expression level in PC9 OsiR cells (left) and HCC827 OsiR cells (right). *n* = 3 biological replicates. (D) Immunofluorescence of p-p62 (S403) condensates in PC9, HCC827, and OsiR cells. (E) The p-p62 (S403) condensates area as a percentage of the cytoplasmic area (quantified across > 9 cells). *n* = 3 biological replicates. (F) Western blot of p62 in p62-KO OsiR and GefR cells transfected with EGFP-tagged p62 WT, ΔPB1, ΔUBA, S403A and S403E. (G) EGFP-p62 condensates in p62-KO GefR cells rescued by wild-type, S403A, or S403E mutated p62. (H) The p62 condensates area as a percentage of cytoplasmic area (quantified across > 9 cells). *n* = 3 biological replicates.Data are mean ± s.e.m.; statistical analysis was performed using two-tailed Student’s *t*-test (E) and one-way ANOVA (B, C, H).

**Fig. S11 S403 phosphorylation drives p62 condensate formation.** (A) Representative fluorescence images of condensates formed by mixing with purified MBP-p62-WT-mCherry, MBP-p62-S403A-mCherry, or MBP-p62-S403E-mCherry at the indicated p62 concentrations in the presence of 100 μM 4×Ub. (B) Quantification of p62 condensate area in a. *n* = 3 biological replicates. (C) Representative fluorescence images of condensates formed by mixing with the indicated concentrations of 4×Ub in the presence of 50 μM MBP-p62-WT-mCherry, 50 μM MBP-p62-S403A-mCherry, or 50 μM MBP-p62-S403E-mCherry. (D) Quantification of p62 condensate area in c. *n* = 3 biological replicates. Data are mean ± s.e.m.; statistical analysis was performed using one-way ANOVA (B, D).

**Fig. S12 TBK1 promotes p62 condensate formation in EGFR-TKI-resistant lung cancer cells.** (A) Immunofluorescence of p62 condensates in GefR cells treated with Gefitinib alone or combination with GSK8612. (B) The p62 condensates area as a percentage of cytoplasmic area (quantified across > 9 cells). n = 3 biological replicates. (C) Immunofluorescence of p62 condensates in OsiR cells transfected with negative control siRNA or two independent TBK1-targeting siRNAs. (D) The p62 condensates area as a percentage of cytoplasmic area (quantified across > 9 cells). n = 3 biological replicates. (E) Western blot of TBK1 overexpression in OsiR cells. (F) Immunofluorescence of p62 condensates in PC9 or HCC827 cells transfected with control or TBK1-overexpression constructs. (G) The p62 condensates area as a percentage of cytoplasmic area (quantified across > 9 cells). n = 3 biological replicates. Data are mean ± s.e.m.; statistical analysis was performed using two-tailed Student’s t-test (B, G) and one-way ANOVA (D).

**Fig. S13 p62 S403 phosphorylation promotes EGFR-TKI resistance in lung cancer cells.** (A) CellTiter-Glo assay of cell viability in p62-KO GefR cells rescued by either wild-type or point-mutated p62 (p62-S403E, p62-S403A), after 72 h treatment of Gefitinib. n = 3 biological replicates. (B) IC_50_ values of Gefitinib in GefR cells. n = 3 biological replicates. (C) Western blot of p62-KO in PC9 or HCC827 cells. (D) CellTiter-Glo assay of cell viability in p62-KO PC9 (left) or HCC827 (right) cells rescued by either wild-type or point-mutated p62 (p62-S403E, p62-S403A), after 72 h treatment of Osimertinib. n = 3 biological replicates. (E) IC_50_ values of Osimertinib in PC9 or HCC827 cells. n = 3 biological replicates. Data are mean ± s.e.m.; statistical analysis was performed using one-way ANOVA(B, E).

**Fig. S14 GSK8612 reduces p62 condensation and restores sensitivity of EGFR-TKI-resistant lung cancer to Gefitinib treatment.** (A) CellTiter-Glo assay of cell viability in GefR cells after 72 h treatment of Gefitinib alone or in combination with GSK8612 at the indicated concentrations.n = 3 biological replicates. (B) IC_50_ values of EGFR-TKIs alone, GSK8612 alone, or their combination in EGFR-TKI-resistant cells. *n* = 3 biological replicate. (C) CI values in PC9 GefR cells were calculated using CompuSyn software based on CellTiter-Glo viability data and plotted as a function of Fa. (D) CellTiter-Glo assay of cell viability in p62-KO OsiR cells after 72 h treatment of Osimertinib alone or in combination with GSK8612 at the indicated concentrations.n = 3 biological replicates. (E) IC_50_ values of Osimertinib alone, GSK8612 alone, or their combination in OsiR cells. n = 3 biological replicate. (F) Colony formation assay of GefR cells after treatment with Gefitinib, GSK8612 alone or in combination. (G) Quantify the colony formation. *n* = 3 technical replicates. (H) The weight of the nude mice was measured on the indicated days. *n* = 5 biological replicates. Data are mean ± s.e.m.; statistical analysis was performed using one-way ANOVA followed(B, E) and two-way ANOVA followed(G).

**Fig. S1 Establishment of EGFR-TKI-resistant lung cancer cell lines.**

**Fig. S2 p62 condensates contribute to Gefitinib resistance in PC9 GefR cells.**

**Fig. S3 The p62 protein is important to EGFR-TKI resistance in lung cancer.**

**Fig. S4 EGFR-TKI-resistant cells exhibit enhanced autophagic flux and lysosomal activation.**

**Fig. S5 Compounds that inhibit or promote p62 condensate formation.**

**Fig. S6 Increased TBK1-p62 interaction accompanies EGFR-TKI resistance.**

**Fig. S7 TBK1 knockdown sensitizes EGFR-TKI-resistant cells to EGFR-TKIs independently of IRF3/IRF7.**

**Fig. S8 p62 S349/S403 phosphorylation is elevated in EGFR-TKI-resistant-cells.**

**Fig. S9 GSK8612 dose-dependently reduces p62 S403 phosphorylation while sparing S349.**

**Fig. S10 TBK1-dependent p62 phosphorylation drives p62 condensates formation in EGFR-TKI-resistant cells.**

**Fig. S11 p62 S403 phosphorylation drives p62 condensate formation**

**Fig. S12 TBK1 promotes p62 condensate formation in EGFR-TKI-resistant lung cancer cells.**

**Fig. S13 p62 S403 phosphorylation promotes EGFR-TKI resistance in lung cancer cells.**

**Fig. S14 GSK8612 reduces p62 condensation and restores sensitivity of EGFR-TKI-resistant lung cancer to Gefitinib treatment.**

**Supplementary Table 1 Patient information for biopsy specimens.**

**Supplementary video 1 Representative time-lapse fluorescence images of PC9 cells transfected with p62-EGFP.**

**Supplementary video 2 Representative time-lapse fluorescence images of PC9 OsiR cells transfected with p62-EGFP.**
